# Tomato PYR/PYL/RCAR abscisic acid receptors show high expression in root, differential sensitivity to the abscisic acid agonist quinabactin, and the capability to enhance plant drought resistance

**DOI:** 10.1093/jxb/eru219

**Published:** 2014-05-26

**Authors:** Miguel González-Guzmán, Lesia Rodríguez, Laura Lorenzo-Orts, Clara Pons, Alejandro Sarrión-Perdigones, Maria A. Fernández, Marta Peirats-Llobet, Javier Forment, Maria Moreno-Alvero, Sean R. Cutler, Armando Albert, Antonio Granell, Pedro L. Rodríguez

**Affiliations:** ^1^Instituto de Biología Molecular y Celular de Plantas, Consejo Superior de Investigaciones Cientificas-Universidad Politecnica de Valencia, ES-46022 Valencia, Spain; ^2^Departamento de Cristalografía y Biología Estructural, Instituto de Química Física ‘Rocasolano’, CSIC, Serrano 119, E-28006 Madrid, Spain; ^3^Department of Botany and Plant Sciences, Center for Plant Cell Biology, University of California, Riverside, CA 92521, USA

**Keywords:** Abscisic acid (ABA), drought resistance, tomato ABA receptor, tomato clade A PP2C.

## Abstract

Abscisic acid (ABA) plays a crucial role in the plant’s response to both biotic and abiotic stress. Sustainable production of food faces several key challenges, particularly the generation of new varieties with improved water use efficiency and drought tolerance. Different studies have shown the potential applications of *Arabidopsis* PYR/PYL/RCAR ABA receptors to enhance plant drought resistance. Consequently the functional characterization of orthologous genes in crops holds promise for agriculture. The full set of tomato (*Solanum lycopersicum*) PYR/PYL/RCAR ABA receptors have been identified here. From the 15 putative tomato ABA receptors, 14 of them could be grouped in three subfamilies that correlated well with corresponding *Arabidopsis* subfamilies. High levels of expression of *PYR/PYL/RCAR* genes was found in tomato root, and some genes showed predominant expression in leaf and fruit tissues. Functional characterization of tomato receptors was performed through interaction assays with *Arabidopsis* and tomato clade A protein phosphatase type 2Cs (PP2Cs) as well as phosphatase inhibition studies. Tomato receptors were able to inhibit the activity of clade A PP2Cs differentially in an ABA-dependent manner, and at least three receptors were sensitive to the ABA agonist quinabactin, which inhibited tomato seed germination. Indeed, the chemical activation of ABA signalling induced by quinabactin was able to activate stress-responsive genes. Both dimeric and monomeric tomato receptors were functional in *Arabidopsis* plant cells, but only overexpression of monomeric-type receptors conferred enhanced drought resistance. In summary, gene expression analyses, and chemical and transgenic approaches revealed distinct properties of tomato PYR/PYL/RCAR ABA receptors that might have biotechnological implications.

## Summary

Chemical and transgenic approaches can activate ABA signalling via crop PYR/PYL ABA receptors; quinabactin can selectively activate tomato ABA receptors, and overexpression of monomeric-type receptors confers enhanced plant drought resistance.

## Introduction

Drought, high salinity, and cold have adverse effects on plant growth and seed production. Abscisic acid (ABA)-induced changes play a central role among the various biochemical and physiological processes required to acquire abiotic stress tolerance (Verslues *et al.*, 2006). Thus, in order to maintain water, ABA promotes stomatal closure through the control of membrane transport systems (Osakabe *et al.*, 2014). On the other hand, shoot growth is inhibited whereas the root growth rate is maintained to gain access to water (Sharp *et al.*, 2004; Des Marais *et al.*, 2012). Gene expression is widely regulated by ABA and, as a result, genes encoding proteins involved in protection and damage repair are up-regulated, such as late embryogenesis abunant (LEA)/dehydrins, reactive oxygen species (ROS) scavengers, or osmolyte biosynthetic enzymes (Verslues *et al.*, 2006). Extensive knowledge of ABA perception and signal transduction has emerged in recent years in *Arabidopsis thaliana* (Cutler *et al.*, 2010). PYR/PYL/RCAR (PYRABACTIN RESISTANCE1/PYR1-LIKE/REGULATORY COMPONENTS OF ABA RECEPTORS) receptors perceive ABA intracellularly and, as a result, form ternary complexes with clade A protein phosphatase type 2Cs (PP2Cs), thereby inactivating them (Ma *et al.*, 2009; Park *et al.*, 2009; Santiago *et al.*, 2009*a*; Umezawa *et al.*, 2009; Vlad *et al.*, 2009; Nishimura *et al.*, 2010). This allows the activation of downstream targets of the PP2Cs, such as the sucrose non-fermenting 1-related subfamily 2 (SnRK2s) protein kinases, namely SnRK2.2/D, 2.3/I, and 2.6/OST1/E, which are key players in the regulation of the transcriptional response to ABA and stomatal aperture (Cutler *et al.*, 2010; Finkelstein, 2013). PP2Cs also dephosphorylate other classes of kinases or kinase-regulated proteins (Finkelstein, 2013; Rodrigues *et al.*, 2013). According to the oligomeric nature of the apo receptors, they can be classified in two major classes: dimeric (PYR1 and PYL1–PYL3) or monomeric (PYL4–PYL10, except the untested PYL7) (Dupeux *et al.*, 2011*a*; Hao *et al.*, 2011). Upon ligand binding, dimeric receptors dissociate to make available the PP2C interaction surface (Dupeux *et al.*, 2011*a*). In particular, PYL3 suffers a severe *cis*- to *trans*-dimer transition by a protomer rotation of 135 º to facilitate subsequent dissociation (Zhang *et al.*, 2012). Monomeric receptors are able to interact with PP2Cs in the absence of ABA; however, the presence of ABA is required for a major inhibition of PP2Cs when protection of phosphorylated protein substrates by ABA receptors is evaluated (Antoni *et al.*, 2012; Pizzio *et al.*, 2013).

Several biochemical and structural studies of apo/ABA-bound PYLs and ternary receptor–ABA–phosphatase complexes have provided the molecular details of the ABA perception and signalling mechanism (Melcher *et al.*, 2009; Miyazono *et al.*, 2009; Santiago *et al.*, 2009*b*; Nishimura *et al.*, 2009; Yin *et al.*, 2009). Two loops located between the β3–β4 and β5–β6 sheets control the access of the ABA molecule to the ABA-binding pocket. These loops generate an open conformation of the ligand-binding pocket in the apo receptors. In response to ABA, conformational changes occur in these loops that serve as a gate and latch to stabilize the closed conformation of the ABA-bound receptor. A conserved serine residue flips out of the β3–β4 loop and inserts into the phosphatase catalytic site, blocking access of potential substrates. The mechanism of PP2C inhibition is further explained by the structure of the ternary receptor–ABA–phosphatase complex. In particular, a conserved tryptophan residue located in a β-hairpin of PP2C establishes contact with the gate and latch loops and indirectly with ABA’s ketone group through a hydrogen bond mediated by a critical water molecule (Melcher *et al.*, 2009; Miyazono *et al.*, 2009; Yin *et al.*, 2009; Dupeux *et al.*, 2011b). As a result of this gate–latch–lock mechanism and PP2C interaction, the ternary complex shows high affinity for ABA binding (*K*_d_ between 20nM and 40nM) (Ma *et al.*, 2009; Santiago *et al.*, 2009a).

Different works have shown the potential applications of *Arabidopsis* PYR/PYL receptors to enhance plant drought resistance, through either genetic engineering or chemical approaches (Santiago *et al.*, 2009a; Saavedra *et al.*, 2010; Mosquna *et al.*, 2011; Cao *et al.*, 2013; Okamoto *et al.*, 2013; Pizzio *et al.*, 2013). Thus, through overexpression of either wild-type, constitutively active receptors or mutated versions that enhance ABA-dependent inhibition of PP2Cs, enhanced drought resistance could be conferred to *Arabidopsis* plants (Santiago *et al.*, 2009a; Saavedra *et al.*, 2010; Mosquna *et al.*, 2011; Pizzio *et al.*, 2013). On the other hand, chemicals acting as ABA agonists have proved to be effective for similar purposes (Cao *et al.*, 2013; Okamoto *et al.*, 2013). Given the physiological and practical implications of the ABA signalling pathway in agriculture, it is expected that similar approaches could be implemented in crops. Identification of core components of the ABA signalling pathway taking advantage of the knowledge generated in *Arabidopsis* is now possible (Ben-Ari, 2012). Thus, since the discovery of the PYR/PYL/RCAR ABA receptor family in *Arabidopsis*, several reports have described orthologous genes in commercial crops, such as tomato (Sun *et al.*, 2011), strawberry (Chai *et al.*, 2011), rice (Kim *et al.*, 2012), grape (Boneh *et al.*, 2012), sweet orange (Romero *et al.*, 2012), and soybean (Bai *et al.*, 2013). Recently, overexpression of *OsPYL5* in the monocot rice was shown to confer enhanced drought tolerance (Kim *et al.*, 2014). In this work, a comprehensive identification of putative PYR/PYL/RCAR receptors was carried out in the dicot tomato and different studies were performed to validate their function and to show that they encode functional ABA receptors in plant cells. As a result, it was found that they inhibited tomato clade A PP2Cs in an ABA-dependent manner and some of them could be activated by the ABA agonist quinabactin (QB), which induced tomato stress-responsive genes. The overexpression in *Arabidopsis* of two tomato receptors from the monomeric subgroups AtPY4-6 and AtPYL7-10 conferred enhanced drought resistance, whereas overexpression of a tomato dimeric receptor from the subgroup AtPYL1 failed to confer this phenotype.

## Materials and methods

### Plant material and growth conditions

*Arabidopsis thaliana* and *Solanum lycopersicum* (cv. Moneymaker) plants were routinely grown under greenhouse conditions (40–50% relative humidity) in pots containing a 1:3 vermiculite–soil mixture. For *Arabidopsis* plants grown under growth chamber conditions, seeds were surface sterilized by treatment with 70% ethanol for 20min, followed by commercial bleach (2.5% sodium hypochlorite) containing 0.05% Triton X-100 for 10min, and, finally, four washes with sterile distilled water. Stratification of the seeds was conducted in the dark at 4 ºC for 3 d. Then, seeds were sown on Murashige and Skoog (MS) plates composed of MS basal salts, 0.1% 2-[*N*-morpholino]ethanesulphonic acid, 1% sucrose, and 1% agar. The pH was adjusted to 5.7 with KOH before autoclaving. Plates were sealed and incubated in a controlled-environment growth chamber at 22 ºC under a 16h light, 8h dark photoperiod at 80–100 μE m^–2^ s^–1^. Tomato seeds were surface sterilized by treatment with commercial bleach (2.5% sodium hypochlorite) containing 0.05% Tween-20 for 30min and four washes with sterile distilled water. MS plates for tomato seeds contained 0.5× MS salts.

### Microarray analysis

Fruits at breaker stage were harvested from *S. lycopersicum* (‘Moneymaker’ and ‘Microtom’) and *S. pimpinellifolium* (‘TO-937’) plants. Pericarp and epidermis were excised manually with a sterile scalpel, frozen, and ground with liquid nitrogen to a fine powder. At least three biologically replicated samples for RNA isolation were prepared from each genotype and tissue from three or more pooled fruits. RNA was extracted from pericarp with the modified cetyltrimethylammonium bromide (CTAB) method (Powell *et al.*, 2012) and from epidermis with the Trizol Reagent (Invitrogen). The RNA clean up protocol was done with the RNA Plant Mini Kit (Qiagen). The RNA pellet was resuspended in nuclease-free water. Samples of total RNA were checked for integrity and quality using an Agilent Bioanalyzer (Agilent Technologies). The three biologically replicated RNA samples were amplified, labelled, and hybridized to the 34K gene EUTOM3 Exon array (https://bioinformatics.psb.ugent.be/gdb/solanum) according to the manufacturer’s instructions (Affymetrix) at Unitat Central d’Investigació (Universitat de Valencia, Spain) as described in Powell *et al.* (2012). Data were pre-processed and analysed using Partek Genomic Suite software v6.6 (Partek Inc.) with the probes matching only once with the ITAG annotation 2.30. The configuration consisted of a pre-background adjustment for GC content, robust multiarray analysis for background correction, quantile normalization, and probe set summarization using median polishing (Irizarry *et al.*, 2003). Library files were eutom3gene_v2_ucprobes.cdf and the annotation file version was eutom3-annotation-per-scaffold-modif.txt which represents 30 000 tomato genes.

### Yeast two hybrid (Y2H) assays

The full-length coding sequences of Sl08g076960, Sl06g061180, Sl09g015380, Sl06g050500, Sl03g095780, Sl12g055990, and Sl03g007310 ABA receptors as well as Sl05g052980 PP2C were amplified by PCR from tomato leaf/fruit cDNA and cloned into the pCR8/GW/TOPO entry vector (Invitrogen). An N-terminal deleted version (ΔN) of Sl12g096020 PP2C was amplified from tomato leaf/fruit cDNA using primers that amplify the catalytic PP2C core (amino acid residues 178–509, ΔN Sl12g096020). All the primers used in this work are listed in Supplementary Table S1 available at *JXB* online. Appropriate restriction sites were introduced in some primers to allow the subsequent cloning steps, and all constructs were verified by DNA sequencing. Tomato ABA receptors were fused by Gateway recombination to the GAL4 DNA-binding domain (GBD) in pGBKT7GW. As preys, a set of *Arabidopsis* clade A PP2Cs fused to the GAL4 activation domain (GAD) in the pGADT7 vector was used (Lackman *et al.*, 2011; Antoni *et al.*, 2012). Tomato Sl05g052980 and ΔN Sl12g096020 PP2Cs were fused to the GAD in the pGADT7GW vector. Protocols for Y2H assays were similar to those described previously (Saez *et al.*, 2008).

### Purification of recombinant proteins

Sl06g050500, Sl03g095780, Sl12g055990, and Sl03g007310 coding sequences were cloned in pCR8/GW/TOPO, excised using *Nco*I/*Eco*RI double digestion, and subcloned into pETM11. Sl09g015380, Sl08g076960, and Sl06g061180 coding sequences have either *Eco*RI or *Nco*I internal restriction sites and were subcloned using a different strategy. Coding sequences of Sl08g076960 and Sl09g015380 were excised using *Nco*I/*Hin*dIII and *Nco*I/*Bam*HI double digestion, respectively, and subcloned into pETM11, whereas Sl06g061180 was excised using *Eco*RI digestion and subcloned into pET28a. *Escherichia coli* BL21 (DE3) cells transformed with the corresponding pET28a/pETM11 construct were grown in 50ml of Luria–Bertani medium supplemented with 50 μg ml^–1^ kanamycin to an optical density at 600nm of 0.6–0.8. Then, 1mM isopropyl-β-d-thiogalactopyranoside (IPTG) was added, and the cells were harvested 3h after induction and stored at –80 ºC before purification. The pellet was resuspended in 2ml of HIS buffer (50mM TRIS-HCl, pH 7.6, 250mM KCl, 10% glycerol, 0.1% Tween-20, and 10mM mercaptoethanol), and the cells were sonicated in a Branson sonifier. A cleared lysate was obtained after centrifugation at 14 000 *g* for 15min, and it was diluted with 2 vols of HIS buffer. The protein extract was applied to a 0.5ml nickel–nitrilotriacetic acid (Ni-NTA) agarose column, and the column was washed with 10ml of HIS buffer supplemented with 20% glycerol and 30mM imidazole. Bound protein was eluted with HIS buffer supplemented with 20% glycerol and 250mM imidazole.

In order to obtain enough protein for size exclusion chromatography (SEC) analysis, 8ml of an overnight culture were subcultured into 800ml of fresh 2TY broth (16g of Bacto tryptone, 10g of yeast extract, 5g of NaCl per litre of solution) plus kanamycin (50 μg ml^–1^). Protein expression was induced with 0.3mM IPTG, and the cells were harvested after overnight incubation at 16 ºC. Pellets were resuspended in 25mM TRIS-HCl pH 8.0, 200mM NaCl, 50mM imidazole, 5mM β-mercaptoethanol, and disrupted by sonication. After centrifugation for 40min at 40 000 *g*, the clear supernatant was filtered (pore diameter 0.45 μm; Millipore Corporation, Bedford, MA, USA). The 6His-tagged proteins were purified using Ni-NTA agarose (Qiagen) according to the manufacturer’s instructions. Proteins were eluted with the following elution buffer: 25mM TRIS-HCl pH 8.0, 200mM NaCl, 500mM imidazole, 5mM β-mercaptoethanol, and cleaved with *Tobacco etch virus* (TEV) protease (1:100). Proteins 8g076960, 6g061180, and 6g050500 were concentrated to 10mg ml^–1^ and 12g055990 was concentrated to 0.7mg ml^–1^. Finally, each purified protein was subjected to gel filtration using a prep grade Superdex200 10/30 (Amersham Biosciences Limited, UK) previously equilibrated with 25mM TRIS-HCl pH 8.0, 200mM NaCl, 5mM β-mercaptoethanol. Approximately 1mg of 8g076960, 6g061180, or 6g050500 was loaded onto the column, whereas 12g055990 was difficult to solubilize and only 0.1mg was loaded.

### PP2C activity assays

Phosphatase activity was measured using the RRA(phosphoT)VA peptide as substrate, which has a *K*_m_ of 0.5–1 μM for eukaryotic PP2Cs (Donella *et al.*, 1990). Assays were performed in a 100 μl reaction volume containing 25mM TRIS-HCl, pH 7.5, 10mM MgCl_2_, 1mM dithiothreitol (DTT), 25 μM peptide substrate, and 0.5 μM PP2C. When indicated, PYR/PYL/RCAR recombinant proteins and ABA or QB (Life Chemicals) were included in the PP2C activity assay. ABA and QB concentrations were 0.1, 0.5, 1, 5, 10, and 50 μM. After incubation for 60min at 30 ºC, the reaction was stopped by the addition of 30 μl of molybdate dye (Baykov *et al.*, 1988), and the absorbance was read at 630nm with a 96-well plate reader. Appropriate controls including dimethylsulphoxide (DMSO) and buffer HIS were included.

### ABA and QB treatment of tomato seedlings

Ten-day-old tomato seedlings (cv. Moneymaker) were mock treated or treated with 10 μM ABA or QB for 3h. Total RNA was extracted using a NucleoSpin RNA plant kit. Synthesis of cDNA and quantitaive real-time PCR (qRT-PCR) analyses were performed as described (Saez *et al.*, 2006). Amplification of the ABA- and stress-responsive *Sl02g084850*, *Sl06g067980*, and *Sl06g019170* genes was done using the primers described in Supplementary Table S1 at *JXB* online. Expression was normalized using the values obtained with *Sl06g009970* (*SlEF1a*).

### Generation of transgenic lines

Sl06g050500, Sl03g007310, and Sl08g076960 coding sequences in the pCR8/GW/TOPO entry clone were recombined by LR reaction into the Gateway-compatible ALLIGATOR2 vector (Bensmihen *et al.*, 2004). The ALLIGATOR2 vector drives expression of the recombined gene under control of the *Cauliflower mosaic virus* (CaMV) 35S promoter and introduces a triple haemagglutinin (HA) epitope at the N-terminus of the encoded protein. Selection of transgenic lines is based on the visualization of green fluorescent protein (GFP) in seeds, whose expression is driven by the specific seed promoter At2S3. The ALLIGATOR2 constructs were transferred to *Agrobacterium tumefaciens* C58C1 (pGV2260) (Deblaere *et al.*, 1985) by electroporation and used to transform Columbia wild type by the floral dip method. T_1_ transgenic seeds were selected based on GFP visualization and sown in soil to obtain the T_2_ generation. Homozygous T_3_ progeny was used for further studies, and expression of HA-tagged protein was verified by immunoblot analysis using anti-HA-peroxidase (Roche).

### Seed germination assays

After surface sterilization of the tomato seeds, ~100 seeds were sown on 0.5× MS plates lacking (control plates) or supplemented with either 1 μM or 10 μM ABA or QB. Seeds were germinated in the dark at 23 ºC for 3 d. In order to score seed germination, radical emergence was analysed at 72h after sowing. Since QB was dissolved in DMSO, control MS plates for QB experiments were supplemented with 0.1% DMSO.

### Root growth assays

*Arabidopsis* seedlings were grown on vertically oriented MS plates for 3 d. Afterwards, 20 plants were transferred to new MS plates lacking or supplemented with the indicated concentrations of ABA. The plates were scanned on a flatbed scanner after 10 d to produce image files suitable for quantitative analysis of root growth using the NIH Image software ImageJ v1.37.

### Drought stress and water status measurement

Plants grown under greenhouse conditions (10 individuals per experiment, three independent experiments) were grown under normal watering conditions for 15 d and then subjected to drought stress by stopping irrigation for 20 d. Next, watering was resumed and the survival rate was calculated after 3 d by counting the percentage of plants that had more than four green leaves. Photographs were taken at the start of the experiment (day 0), after 16 d and 20 d of drought, and 3 d after re-watering. The relative water content (RWC) of the plants was measured in rosette leaves at 11, 14, and 17 d. Samples of 10 leaves from five plants were collected and their fresh weight (FW) was obtained. Leaves were then floated for 3h on demineralized water and weighed again in order to obtain their turgid weight (TW). Finally, leaves were dried for 16h at 70 °C and weighed to obtain the dry weight (DW). The RWC was calculated as (FW–DW/TW–DW)×100 and each measurement was made in triplicate.

## Results

### The tomato genome encodes 15 putative PYR/PYL/RCAR ABA receptors

**Fig. 1.**
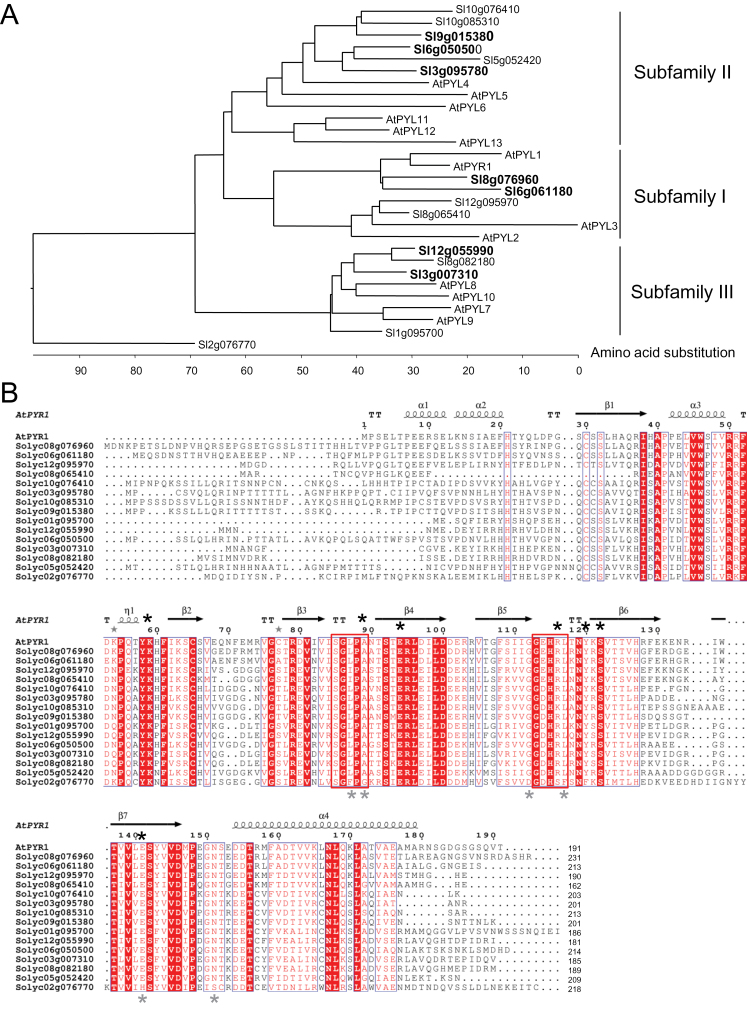
Cladogram and amino acid sequence alignment of tomato PYR/PYL ABA receptors. (A) Cladogram of the multiple sequence alignment of tomato and *Arabidopsis* PYR/PYL receptors, indicating three major subfamilies and the ungrouped 2g076770. Those tomato receptors further described in the text are in bold face. (B) Sequence and secondary structure alignment of tomato PYR/PYL ABA receptors and *Arabidopsis* PYR1 protein. The predicted secondary structure of the tomato proteins was indicated, taking as a model the crystallographic structure of PYR1 (Protein DataBank Code 3K90) and using the Espript interface (http://espript.ibcp.fr/). Boxes indicate the position of the gate and latch loops. Black asterisks mark residues K59, A89, E94, R116, Y120, S122, and E141 of PYR1 involved in ABA binding. Grey asterisks mark conserved residues of the tomato receptor family that differ in tomato 2g076770 protein. (This figure is available in colour at *JXB* online.)

**Fig. 2.**
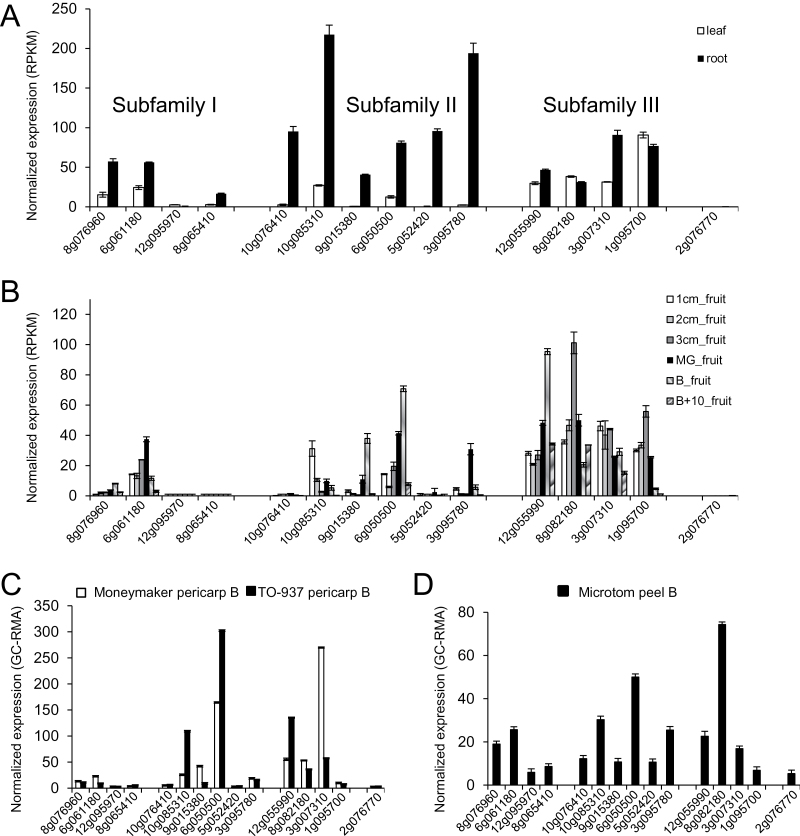
Relative gene expression of tomato ABA receptors in leaf, root, and fruit was determined by RNA-Seq and microarray analysis. (A, B) The transcriptome of the inbred tomato cultivar Heinz 1706 was analysed using Illumina RNA-Seq technology (Tomato genome Consortium, 2012). Data show gene transcription of tomato receptors grouped in three subfamilies and the ungrouped 2g076770 in leaf and root (A) and during fruit development and ripening (B). Significant expression of 2g076770 and 12g095970 was not detected in these tissues. RPKM, reads per kilobase of exon model per million mapped reads; MG, mature green stage; B, breaker stage. (C, D) Relative RNA abundance based on GC-RMA values (GC content–robust multiarray analysis) obtained from the Affymetrix exon tomato microarray (EUTOM3) hybridized with fruit pericarp RNA of Moneymaker and TO-937 (C) and fruit epidermis RNA of the Microtom background (D) at the breaker stage.

A partial analysis of the tomato PYR/PYL/RCAR family was published by Sun *et al.* (2011), leading to the discovery of eight receptors. The analysis has now been extended to the complete tomato genome (Tomato Genome Consortium, 2012) and, as a result, 15 receptors have been identified (Fig. 1A). With the exception of 2g076770, they were distributed in three subfamilies, which matched the corresponding groups from *Arabidopsis* PYR/PYL/RCAR receptors. Since it is possible that biochemical or physiological features already known in *Arabidopsis* receptors might be translated to crop receptors, an attempt was made to correlate tomato receptors with the corresponding groups in *Arabidopsis* (Fig. 1A). On this basis, a new nomenclature is proposed. Thus, in subfamily I, two tomato receptors, 8g076960 and 6g061180, closely related to *Arabidopsis* PYL1/PYR1 (Supplementary Fig. S1 at *JXB* online), and two other receptors, 12g095970 and 8g065410, more closely related to AtPYL2/PYL3, were found. In subfamily II, six tomato receptors related to AtPYL4/PYL5/PYL6 were found and in subfamily III four tomato receptors related to AtPYL7/PYL8/PYL9/PYL10 were found. No close relative for the *Arabidopsis* group AtPYL11/12/13 was found in tomato. Finally, the putative tomato receptor 2g076770 was ungrouped, probably because it lacked key conserved residues of the PYR/PYL/RCAR family, such as the conserved leucine of the β3–β4 and β5–β6 loops and the asparagine residue before the α4-helix (Fig. 1B). Additionally, 2g076770 gene expression could not be detected by RNA-Sequencing (RNA-Seq) analysis in vegetative or fruit tissue (see below Fig. 2); therefore, it remains to be established whether 2g076770 is a functional ABA receptor. In contrast, the key residues of both gate-like and latch-like loops were conserved in the remaining 14 tomato receptors (Fig. 1B).

### Differential gene expression of tomato ABA receptors in leaf, root, and fruit

Relative expression of all tomato genes has recently been reported using Illumina RNA-Seq technology, providing gene expression data for the transcriptome of the inbred tomato cultivar ‘Heinz 1706’ (Tomato Genome Consortium, 2012). Data mining was performed in the RNA-Seq transcriptome for the 15 tomato receptor genes in root, leaf, and six stages of fruit, and additionally a further microarray analysis was performed in other tomato accessions (Fig. 2). The expression pattern of ABA receptors in root and leaf indicates that some members clearly show a higher expression level compared with others (Fig. 2A). Two members from subfamily II, namely 10g085310 and 3g095780, showed the highest transcription level in root, whereas a member from subfamily III—1g095700—was the most transcribed receptor in leaf. In contrast, expression of some receptors was absent in these tissues. For instance, significant expression of 2g076770 and 12g095970 was not detected in either leaf or root (Fig. 2A). On the other hand, it was interesting to detect a high expression of several tomato ABA receptors in root tissue since ABA signalling is required at low water potentials to maintain primary root elongation, to increase root versus shoot biomass partitioning, to regulate root system architecture, and to promote root hydrotropism (Sharp *et al.*, 2004; Des Marais *et al.*, 2012; Duan *et al.*, 2013). RNA-Seq data were also compiled from a tomato fruit development series composed of six fruit stages: 1cm, 2cm, 3cm, mature green, breaker (when colour becomes noticeable), and breaker +10 d. In general, members from subfamily III, for instance 12g055990 and 8g082180, showed high expression levels during fruit development, which suggests a role in this process (Fig. 2B). Additionally, recent studies suggest that ABA might be involved in regulating the onset of fruit ripening through triggering of ethylene biosynthesis and, consequently, ABA content peaks at breaker stages compared with the mature green stage (Zhang *et al.*, 2009; Sun *et al.*, 2011). Some tomato ABA receptors whose expression peaked during breaker stages were identified in subfamily II and III, such as 6g050500 and 12g055990; therefore they are candidate genes to regulate fruit ripening (Fig. 2B).

In order to obtain further data on fruit expression of tomato *PYR/PYL* genes, new studies were performed through microarray expression analysis in fruit pericarp at the breaker stage for cv. Moneymaker and *S. pimpinellifolium* accession TO-937 (Fig. 2C). Both 6g050500 and 12g055990 were among the top three most expressed genes at the breaker stage, although, depending on the cultivar considered, other tomato receptors also appeared to be highly expressed (for instance 3g007310 in Moneymaker and 10g085310 in TO-937). In any case, the comparison between Heinz, Moneymaker, and TO-937 indicates that 6g050500 was highly expressed in all three genetic backgrounds at the breaker stage, which suggests that it could be relevant in the regulation of tomato ripening. ABA receptors of subfamily III may also be important but in an accession-specific manner.

Fruit epidermis might be a putative target of ABA action to minimize fruit water loss, for instance through regulation of cuticle thickness or cuticle-dependent sensing of osmotic stress (Wang *et al.*, 2011), and fruit peel also represents the point of entry for pathogen attack. Different genetic resources in the tomato Microtom background are available, including transgenic lines that modify fruit peel features (Shi *et al.*, 2013). Therefore, it was of interest to obtain gene expression data for ABA receptors in fruit peel. Microarray expression data indicated different receptors from subfamily I, II, and III that were expressed at above average levels in fruit peel and, are, therefore, candidates to regulate ABA action at the fruit epidermis (e.g. 6g050500 and 8g082180; Fig. 2D).

### Tomato ABA receptors interact with *Arabidopsis* and tomato clade A PP2Cs

**Fig. 3.**
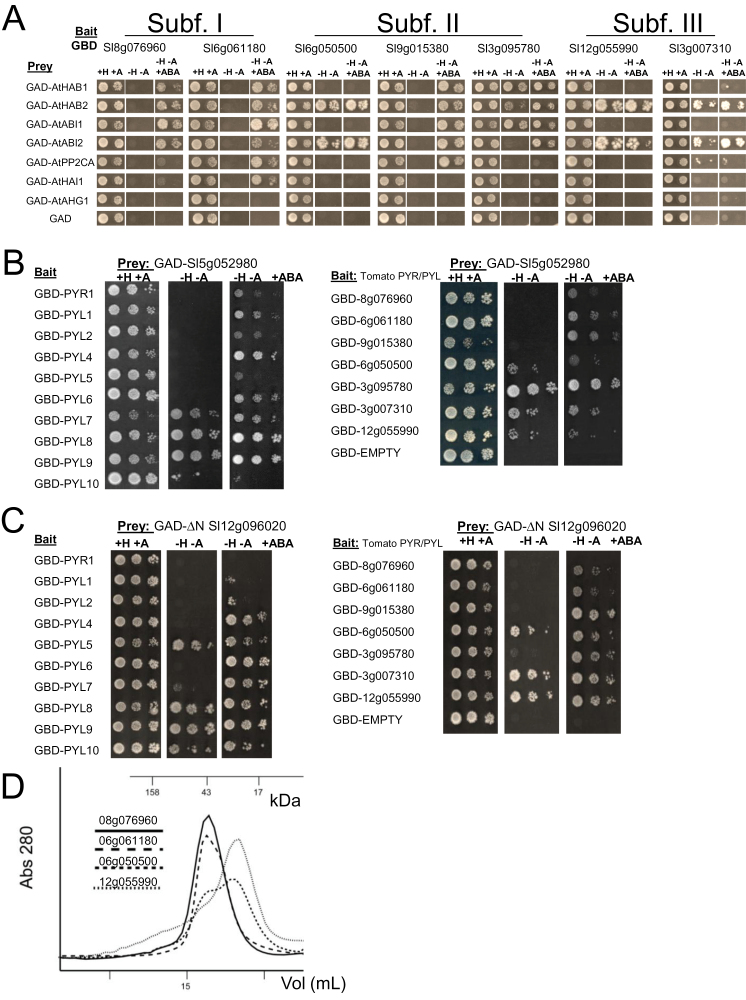
Interactions between PYR/PYL ABA receptors and clade A PP2Cs. Interaction was determined by growth assay on media lacking histidine and adenine (–H, –A), which were supplemented or not with 50 μM ABA (+ABA). Dilutions (10^–1^, 10^–2^, and 10^–3^) of saturated cultures were spotted onto the plates. (A) Interaction of tomato receptors with *Arabidopsis* PP2Cs. (B) Interaction of a tomato PP2CA-like phosphatase (5g052980) and *Arabidopsis* (left) or tomato (right) PYR/PYL ABA receptors. (C) Interaction of a tomato ΔN HAB1-like phosphatase (12g096020) and *Arabidopsis* (left) or tomato (right) PYR/PYL ABA receptors. (D) Elution profiles after size-exclusion chromatography of four tomato ABA receptors in the absence of ABA. The lines show the absorbance recorded at 280nm. Molecular mass markers are indicated in kDa. (This figure is available in colour at *JXB* online.)

A key aspect of receptor function is its ability to interact and inhibit clade A PP2Cs. Both ABA-independent and ABA-dependent Y2H interactions among ABA receptors and PP2Cs have been reported in *Arabidopsis*, which probably reflect the monomeric/dimeric nature of the receptor as well as different *K*_d_s in yeast for particular receptor–phosphatase interactions (Ma *et al.*, 2009; Park *et al.*, 2009; Santiago *et al.*, 2009*a*). ABA-independent interactions can be detected in Y2H assays; however, major inhibition of PP2C activity relies on the presence of ABA (Ma *et al.*, 2009; Park *et al.*, 2009; Santiago *et al.*, 2009*a*). First of all, it was tested whether tomato PYR/PYLs were able to interact with *Arabidopsis* clade A PP2Cs. Tomato members were selected from the three subfamilies and both ABA-independent and ABA-dependent interactions with *Arabidopsis* PP2Cs were found (Fig. 3A). Interestingly, both 8g076960 and 6g061180, which are thought to be dimeric receptors according to their similarity to AtPYL1, showed ABA-dependent interactions with *Arabidopsis* PP2Cs (Fig. 3A). Dimeric receptors occlude their surface of interaction with PP2Cs and require ABA-induced dissociation to form ternary receptor monomer–ABA–phosphatase complexes (Dupeux *et al.*, 2011*a*). Three members from subfamily II were assayed and it was found that two of them showed ABA-independent interactions with some PP2Cs. The third member, 9g015380, is a close relative of AtPYL4 and it showed ABA-dependent interactions with PP2Cs, as was reported previously for AtPYL4 (Lackman *et al.*, 2011). Finally, two members from subfamily III were assayed and ABA-independent interactions with AtHAB2 and AtABI2 were found. The *Arabidopsis* PP2C AHG1 is resistant to inhibition by ABA receptors because it lacks the conserved tryptophan residue required for formation of ternary complexes (Dupeux *et al.*, 2011*b*). Accordingly, this phosphatase did not interact with any tomato receptor, suggesting a similar tryptophan-dependent mechanism for formation of ternary complexes with tomato receptors. Finally, the PP2C HAI1, which shows a more restrictive pattern of interaction with *Arabidopsis* receptors, only interacted with one tomato receptor (Bhaskara *et al.*, 2012).

Next the cDNA of two tomato clade A PP2Cs, 5g052980 and 12g096020, which are close relatives of *Arabidopsis* PP2CA and HAB1, respectively, were cloned (Supplementary Fig. S2 at *JXB* online). The N-terminal deleted version (ΔN) of 12g096020 was used for both Y2H and activity assays, since the N-terminus is dispensable for interaction with ABA receptors (Santiago *et al.*, 2009*a*). 5g052980 and ΔN 12g096020 showed both ABA-dependent and ABA-independent interactions with *Arabidopsis* as well as tomato receptors (Fig. 3B, C). Therefore, a similar mechanism to that described in *Arabidopsis* for receptor–phosphatase interaction seems to operate in tomato. Finally, according to Y2H analyses and sequence similarity, it was predicted that tomato relatives of AtPYL1, such as 8g076960 and 6g061180, might be dimeric receptors whereas members of subfamilies II and III might be monomeric receptors. To test this prediction, SEC analysis was performed for four tomato receptors in the absence of ABA (Fig. 3D). As a result, it was found that 8g076960 and 6g061180 migrated with an estimated molecular mass of 48kDa, which corresponds to a dimeric receptor, and 12g055990 migrated with an estimated molecular mass of 27kDa, which corresponds to a monomeric receptor (predicted molecular masses are 25.6, 23.7, and 25.8kDa, respectively). However, 6g050500 displays a two-peak profile that corresponds to a distribution between monomeric and dimeric species (predicted molecular mass is 24.5kDa). Recent results with the monomeric receptor AtPYL9 indicate that it can form dimers during crystal packing and a minor dimeric form also appears after SEC (Zhang *et al.*, 2013).

### Tomato ABA receptors inhibit both *Arabidopsis* and tomato clade A PP2Cs in an ABA-dependent manner

**Fig. 4.**
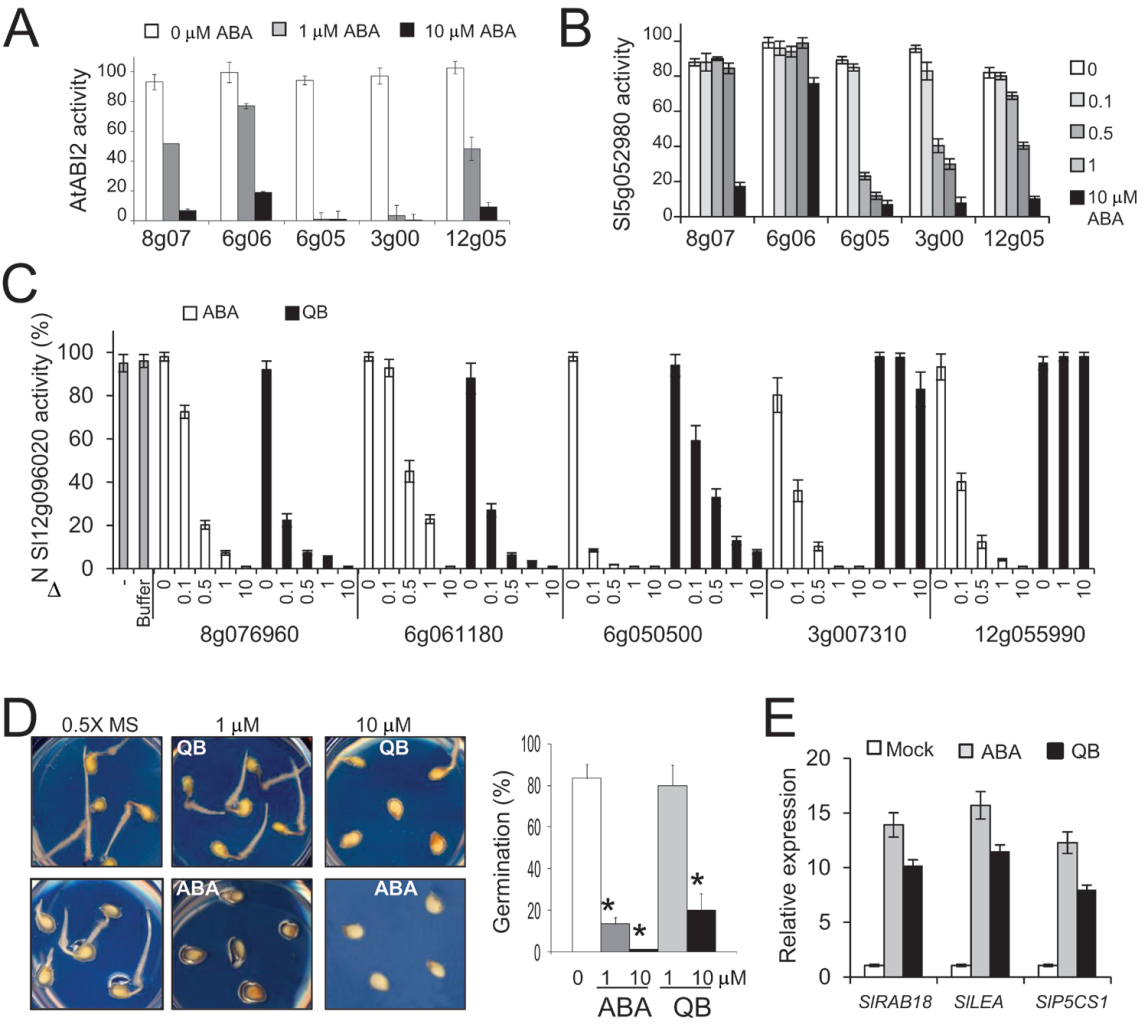
ABA-dependent PP2C inhibition mediated by tomato ABA receptors. PP2C activity was measured *in vitro* using a phosphopeptide substrate in the absence or presence of ABA at a 1:4 ratio of phosphatase:receptor (0.5:2 μM stoichiometry). Data are averages ±SD for three independent experiments. (A) ABA-dependent inhibition of AtABI2 by tomato receptors. Values represent percentage activity compared with 100% in the absence of receptor and ABA. (B) Phosphatase activity of tomato 5g052980 in the presence of tomato receptors. (C) Phosphatase activity of tomato ΔN 12g096020 PP2C in the presence of tomato receptors. PP2C activity was measured in the absence or presence of 0, 0.1, 0.5, 1, or 10 μM ABA or QB. The column labelled as buffer contained an equivalent volume of HIS elution buffer and 0.5% DMSO. (D) Inhibition of tomato seed germination is more sensitive to ABA than QB. Seed germination was scored 72h after sowing. * indicates *P*<0.05 (Student’s *t*-test) when comparing data of plates supplemented with ABA or QB with plates lacking these chemicals. (E) QB treatment induces expression of ABA- and stress-responsive genes. Ten-day-old tomato seedlings were either mock treated or treated with 10 μM ABA or QB for 3h. The histograms indicate the relative induction by ABA or QB treatment of the indicated tomato genes with respect to mock conditions (value 1). (This figure is available in colour at *JXB* online.)

To investigate whether tomato PYR/PYL proteins are actually functional receptors, able to perceive ABA and to inhibit PP2Cs, phosphatase activity was measured using the RRA(phosphoT)VA phosphopeptide as substrate (Fig. 4A–C). To this end, the *Arabidopsis* PP2C ABI2, which showed interaction with all tomato receptors assayed in Y2H assays, and two tomato PP2Cs, 5g052980 and ΔN 12g096020, which are putative orthologous proteins from AtPP2CA and AtHAB1, respectively, were used. It was possible to purify recombinant soluble proteins for five tomato receptors representing the three subfamilies. All of them were able to inhibit phosphatase activity in an ABA-dependent manner, although to different extents. ABI2 and ΔN 12g096020 activity was sensitive to all receptors, whereas 5g052980 activity was hardly affected by 6g061180 and only at 10 μM ABA by 8g076960. Since tomato phosphatases belong to different sub-branches of the clade A PP2C family, these results are in agreement with the differential inhibition by ABA receptors described previously for AtPP2CA and AtHAB1 (Hao *et al.*, 2011; Antoni *et al.*, 2012; Pizzio *et al.*, 2013).

### The ABA agonist quinabactin is selectively perceived by tomato ABA receptors and induces abiotic stress-responsive genes

QB is an ABA-mimicking ligand able to discriminate among the different *Arabidopsis* PYR/PYL receptors, showing preferential activation of dimeric receptors and certain activation of monomeric PYL5 and PYL7 (Cao *et al.*, 2013; Okamoto *et al.*, 2013). QB application in crop plants (soybean, barley, and maize) had ABA-like effects (Okamoto *et al.*, 2013); however, its mechanism of action has not been investigated previously using crop ABA receptors and PP2Cs. Since QB represents a synthetic ABA agonist eliciting both seed and vegetative ABA responses, having the potential to enable plant protection against water stress, its effect was tested on tomato PYR/PYLs. Interestingly, QB inhibited (compared with ABA) ΔN12g096020 phosphatase activity efficiently through the dimeric tomato receptors 8g076960 and 6g061180, and also through 6g050500, although in this latter case less efficiently than ABA (Fig. 4C). 6g050500 belongs to the AtPYL4–AtPYL6 subfamily and therefore shows similarity to the QB-sensitive AtPYL5, which can explain its capacity to perceive QB. In contrast, two tomato receptors that belong to the AtPYL7–AtPYL10 family, namely 3g007310 and 12g055990, were not activated even by 10 μM QB. These results lend support to the selective effect of QB on ABA receptors and provide biochemical evidence that QB can be perceived by dicot crop receptors and inhibit the activity of a crop PP2C. In order to test whether QB has *in vivo* effects on tomato, tomato seed germination was analysed in the presence of the compound (Fig. 4D). QB was able to inhibit germination of tomato seeds, although at a higher concentration compared with ABA. These results suggest that tomato receptors not sensitive to QB are required for full regulation of seed germination or that QB-sensitive receptors are not expressed at high levels during this stage. Alternatively, since QB is less water soluble than ABA, the bioavailability of QB could be lower than that of ABA to inhibit seed germination or follow a less efficient transport system.

Finally, in order to assess the biological activity of QB in tomato seedlings, 10-day-old plants were treated with 10 μM QB or ABA for 3h. The transcriptional levels of ABA- and drought-responsive tomato genes were analysed using qRT-PCR (Fig. 4E). To this end, three tomato genes were selected that showed strong sequence similarity with either *Arabidopsis RESPONSIVE TO ABA 18* (*RAB18*), *LATE EMBRYOGENESIS ABUNDANT* (*LEA*) family, or the *DELTA 1-PYRROLINE-5-CARBOXYLATE SYNTHASE* (*P5CS1*) genes, which were represented by the tomato loci *2g084850*, *6g067980*, and *6g019170*, respectively. The *Arabidopsis* genes have been shown to be induced in response to drought, cold, salinity, and ABA, and therefore are good markers of plant response to these forms of abiotic stress (Saez *et al.*, 2006). The three tomato genes were activated by both ABA and QB treatment, which indicated that ABA signalling was efficiently triggered by the ABA agonist QB in tomato (Fig. 4E).

### Overexpression of tomato monomeric-type ABA receptors in *Arabidopsis* confers enhanced response to ABA and plant drought resistance

**Fig. 5.**
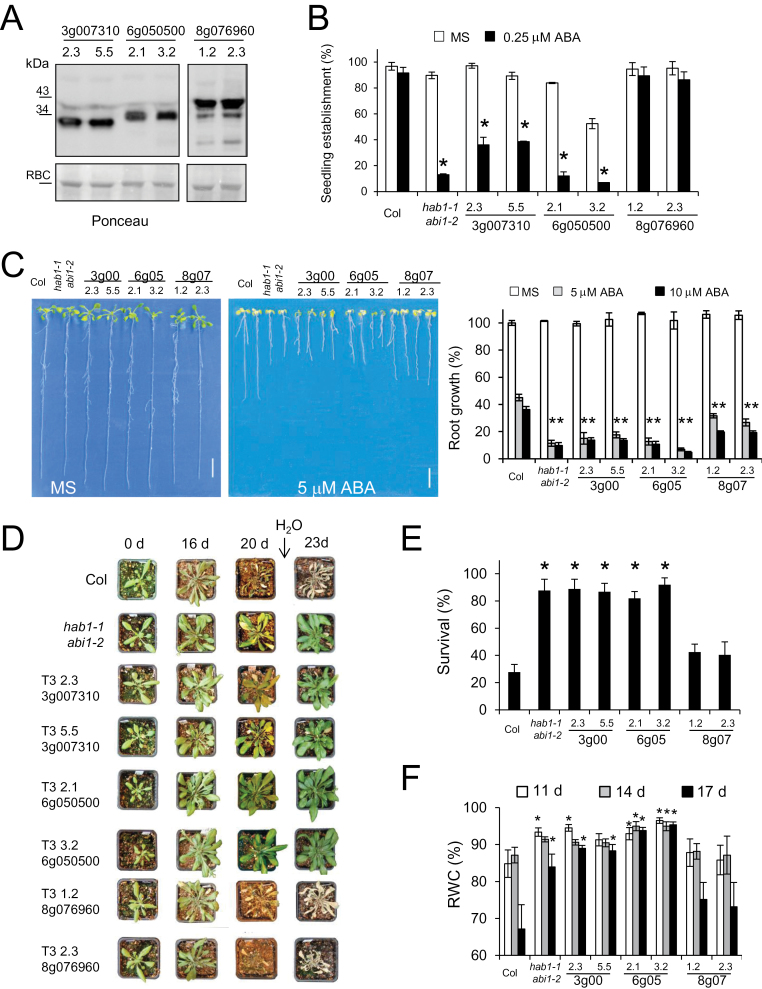
Overexpression of monomeric-type tomato receptors in *Arabidopsis* confers enhanced response to ABA and drought resistance. (A) Immunoblot analysis using antibody against the haemagglutinin (HA) tag shows expression of tomato ABA receptors in 21-day-old seedlings (two independent *Arabidopsis* T_3_ transgenic lines for each tomato receptor). Ponceau staining is shown below. RBC indicates ribulose-1,5-bisphosphate carboxylase. (B) ABA-mediated inhibition of seedling establishment in transgenic lines compared with non-transformed Col plants and the *hab1-1abi1-2* double mutant. * indicates *P*<0.05 (Student’s *t*-test) when comparing data of transgenic lines and the *hab1-1abi1-2* mutant with non-transformed Col plants in the same assay conditions. Approximately 100 seeds of each genotype (three independent experiments) were sown on MS plates lacking or supplemented with 0.25 μM ABA. Seedlings were scored for the presence of both green cotyledons and the first pair of true leaves after 8 d. Values are averages ±SE. (C) Enhanced sensitivity to ABA-mediated inhibition of root growth of transgenic lines and the *hab1-1abi1-2* mutant compared with non-transformed Col plants. Photographs show representative seedlings 10 d after the transfer of 4-day-old seedlings to MS plates lacking or supplemented with 5 μM ABA. Right panel: quantification of ABA-mediated root growth inhibition (values are means ±SE; growth of Col wild type on MS medium was taken as 100%). (D–F) Transgenic lines overexpressing monomeric-type receptors show enhanced drought resistance and survival, and higher RWC compared with non-transformed plants. (D) Two-week-old plants were deprived of water for 20 d and then re-watered. Photographs were taken at the start of the experiment (0-d), after 16 d and 20 d of drought, and 3 d after re-watering. (E) Percentage survival of non-transformed Col, *hab1-1abi1-2*, and transgenic lines 3 d after re-watering. (F) RWC of non-transformed Col, *hab1-1abi1-2*, and transgenic lines after 11, 14, and 17 d of water withdrawal. (This figure is available in colour at *JXB* online.)

Overexpression of some monomeric *Arabidopsis* PYR/PYL receptors is known to enhance ABA response and plant drought resistance (Santiago *et al.*, 2009*a*; Saavedra *et al.*, 2010; Pizzio *et al.*, 2013). In order to investigate whether tomato PYR/PYLs are functional receptors in plant cells, transgenic plants that overexpress HA-tagged versions of either monomeric-type receptors, 6g050500 or 3g007310, or a dimeric receptor, 8g076960, were generated. Expression of HA-tagged tomato PYR/PYLs was verified by immunoblot analysis, and two independent transgenic lines were selected for further analysis (Fig. 5A). Overexpression of tomato monomeric-type receptors in *Arabidopsis* enhanced ABA-mediated inhibition of seedling establishment and root growth compared with non-transformed plants, a phenotype similar to that obtained by a double inactivation of the ABI1 and HAB1 PP2Cs (Fig. 5B, C) (Saez *et al.*, 2006). Interestingly, overexpression of the tomato dimeric receptor did not enhance ABA-mediated inhibition of seedling establishment but enhanced root growth sensitivity to ABA and it also generated partial complementation of the ABA-insensitive phenotype of the 112458 *pyr/pyl* mutant (Fig. 5B, C; Supplementary Fig. S3 at *JXB* online).

Next, drought resistance experiments were performed under greenhouse conditions. Plants were grown under normal watering conditions for 2 weeks and then irrigation was stopped for 20 d (Fig. 5D). After 20 d without watering, non-transformed plants wilted and many rosette leaves yellowed, in contrast to transgenic lines that express tomato monomeric-type PYR/PYLs (Fig. 5D). Interestingly, transgenic lines expressing the tomato dimeric receptor showed a phenotype similar to the wild type (Fig. 5D). Watering was then resumed and survival of the plants was measured after 3 d. A remarkable enhanced survival (40–50%) was found in the drought-resistant *hab1-1abi1-2* double mutant (Saez *et al.*, 2006) and transgenic plants expressing tomato monomeric-type PYR/PYLs compared with non-transformed plants or transgenic lines expressing the dimeric receptor (Fig. 5E). Thus, a clear distinction regarding drought resistance was found between overexpressing tomato monomeric-type or dimeric receptors. During the drought stress experiment, the RWC of the rosette leaves was measured at 11, 14, and 17 d after water withdrawal. Both the *hab1-1abi1-2* double mutant and transgenic lines expressing monomeric-type PYR/PYLs showed higher RWC compared with non-transformed plants or transgenic lines expressing the dimeric receptor (Fig. 5F). Thus, either knocking out clade A PP2Cs or overexpressing tomato monomeric-type receptors leads to plants that experience lower water loss compared with non-transformed plants.

## Discussion

In this work distinct properties of tomato PYR/PYL ABA receptors according to gene expression and biochemical analyses, sensitivity to the ABA agonist QB, and capability to enhance plant drought resistance are revealed. It is demonstrated that both chemical and transgenic approaches can trigger activity of tomato PYR/PYL ABA receptors, leading to inhibition of crop PP2Cs. Thus, the results indicate that chemical treatment with an ABA agonist is effective to activate the ABA signalling pathway in a dicot crop plant, inducing key genes for drought stress response. Inhibition of PP2C activity by either overexpression of ABA receptors or combined insertional mutagenesis has proved to be an efficient approach to enhance plant drought resistance in *Arabidopsis* (Saez *et al.*, 2006; Santiago *et al.*, 2009*a*). The results open the way for similar approaches in tomato given the feasibility of transgenic approaches and the availability of tomato mutant libraries and TILLING platforms (Okabe *et al.*, 2012). According to results obtained here when tomato receptors were introduced into *Arabidopsis* and their effect on AtABI2 inhibition, it seems that ABA receptors can be functionally exchanged among different plants. Therefore, the generation of constitutively active receptors or mutated versions that enhance ABA-dependent inhibition of PP2Cs might be used as a transversal approach to enhance drought resistance in different plants (Mosquna *et al.*, 2011; Pizzio *et al.*, 2013). Since overexpression of monomeric-type tomato receptors in *Arabidopsis* conferred enhanced survival and higher RWC upon drought stress, it will be interesting to check it in tomato plants, through either constitutive or stress-induced expression. Interestingly, overexpression of a dimeric tomato receptor was not effective to enhance *Arabidopsis* drought resistance, which suggests that monomeric crop PYR/PYL ABA receptors might perform better to achieve such a goal. Indeed, in contrast to monomeric *Arabidopsis* PYR/PYL receptors, overexpression of dimeric receptor has not proved to be effective to enhance plant drought resistance, which might reflect structural constraints of dimeric receptors to interact with PP2Cs in the absence of ABA and therefore lack of basal activation of the pathway (Dupeux *et al.*, 2011*a*). In this case, chemical treatment with ABA agonists is an alternative and efficient approach to activate dimeric receptors (Cao *et al.*, 2013; Okamoto *et al.*, 2013).

Tomato is mainly produced in Mediterranean countries, where fresh water availability is a major problem that could be made worse in the event of climate change. When produced in soil-less greenhouses, solution recycling is mandatory and thus any reduction in the water used is relevant for producers. Gene transcription data suggest that a high number of tomato receptors operate in the root and their transcript levels are higher than those in leaves, which seems to support a relevant role for ABA perception and signalling in the root to cope with drought stress and promote a hydrotropic growth response (Sharp and LeNoble, 2002; Antoni *et al.*, 2013). These data are in agreement with a recent transcriptional analysis of *Arabidopsis* genes involved in ABA synthesis and perception, which revealed that whereas genes involved in synthesis show higher levels in shoots than in roots, genes involved in perception show an opposite pattern (Boursiac *et al.*, 2013). On the other hand, some reports have revealed a role for ABA in tomato fruit ripening and cell wall catabolism via regulation of ethylene biosynthesis and major catabolic genes (Zhang *et al.*, 2009; Sun *et al.*, 2012). Therefore, ABA signalling in tomato fruit might also be relevant to regulate fruit texture, shelf life, and water loss through the fruit epidermis (Sun *et al.*, 2012; this work). In summary, both RNA-Seq and microarray expression analyses in tomato have indicated ABA receptors that showed a preferential expression (Sun *et al.*, 2011; Wang *et al.*, 2013; this work). These data together with their functional characterization open the door to a future biotechnological use in order to enhance tomato drought resistance or modify fruit properties regulated through ABA signalling.

*In silico* identification of the ABA signalling core components has been performed in several crops. For instance, the soybean and rice genome encode 23 and 13 putative ABA receptors, respectively (Bai *et al*., 2012; Kim *et al.*, 2012). In general, the nomenclature of ABA receptors in crops has followed a numerical order that lacks correlation with *Arabidopsis* receptors. As a result, current nomenclature in crops makes it difficult to correlate biochemical and physiological properties of crop ABA receptors tentatively with *Arabidopsis* receptors. Since the ABA signalling pathway is universally conserved in land plants (Hauser *et al.*, 2011), it seems sensible to take advantage of knowledge of PYR/PYL receptors in *Arabidopsis*. In this work, a nomenclature is proposed based on ascribing crop receptors to *Arabidopsis* subfamilies of PYR/PYL ABA receptors. This approach is supported by phylogenetic studies showing that PYR/PYL receptors can be grouped in three major clades and it allows tentative prediction of some receptor properties (Hauser *et al.*, 2011). Indeed, it was possible to take advantage of this knowledge to infer some properties exhibited by tomato receptors regarding their oligomeric nature, inhibition of PP2C activity, and sensitivity to QB, for instance. Subfamily I corresponded to *Arabidopsis* dimeric receptors and, indeed, SEC analysis of two tomato members (closely related to AtPYL1) confirmed their dimeric nature. In agreement, Y2H interaction assays showed ABA-dependent interactions for dimeric receptors and PP2Cs, which presumably reflects the requirement for ABA-induced dissociation prior to interaction with the phosphatase. Subfamily II and III corresponded to AtPYL4–AtPYL 6 and AtPYL7–AtPYL 10 groups, respectively. SEC analysis of one tomato representative member of the AtPYL7– AtPYL10 group revealed a similar monomeric nature, whereas the tomato members of the AtPYL4–AtPYL6 group showed an elution profile that might be explained as a monomeric–dimeric mixture. It is possible that the high protein concentration present in the injected sample for SEC analysis might have promoted such equilibrium, as was described recently for the monomeric receptor AtPYL9 (Zhang *et al.*, 2013).

Y2H analyses performed among tomato ABA receptors and either *Arabidopsis* or tomato PP2Cs revealed both ABA-independent and ABA-dependent interactions (Fig. 3). However, major inhibition of PP2C activity by tomato ABA receptors was ABA dependent (Fig. 4). Therefore, these results suggest that the formation of stable ternary receptor–ABA–phosphatase complexes is required to achieve a major effect on the activation of tomato PP2C downstream targets. Clade A PP2Cs constitute a hub for regulation of different environmental responses, allowing the integration of stress signalling pathways into a coordinated response (Rodrigues *et al.*, 2013). A fine tuning of their activity can be achieved by the selective or differential inhibition of PP2C activity carried out by ABA receptors, which was confirmed in the two tomato PP2Cs analysed in this work. Finally, it was also found that the ABA agonist QB was selective for some tomato receptors and promoted both *in vitro* inhibition of a tomato clade A PP2C and *in vivo* inhibition of tomato seed germination. These results, taken together with previous data from Okamoto *et al.* (2013), indicate that the ABA signalling pathway can be activated in crops by chemicals mimicking ABA action. Thus, chemical treatment of tomato seedlings with QB promoted expression of ABA- and stress-responsive genes. For instance, *P5CS1*, which encodes a key enzyme for proline biosynthesis and osmotic adjustment under drought stress, was efficiently induced by QB treatment (Fig. 4E). In summary, both chemical and transgenic approaches based on PYR/PYL ABA receptors might be effective to cope with water stress in tomato.

## Supplementary data

Supplementary data are available at *JXB* online

Figure S1. Amino acid sequence alignment of AtPYL1 and putative tomato orthologous ABA receptors.

Figure S2. Amino acid sequence alignment of AtHAB1, AtPP2CA, and putative tomato orthologous PP2Cs.

Figure S3. Complementation of the 112458 *pyr/pyl* mutant by tomato PYR/PYL receptors.

Table S1. List of oligonucleotides used in this work.

Supplementary Data

## Abbreviations:

ABA: abscisic acid
PP2C: protein phosphatase type 2C
PYR/PYL/RCAR: PYRABACTIN RESISTANCE1/PYR1-LIKE/REGULATORY COMPONENTS OF ABA RECEPTORS
QB: quinabactin.
