# Supplementary material for: Tomato PYR/PYL/RCAR abscisic acid receptors show high expression in root, differential sensitivity to the abscisic acid agonist quinabactin, and the capability to enhance plant drought resistance
Source: J Exp Bot. 2014 May 26;65(15):4451–64. doi: 10.1093/jxb/eru219 (PMC4112642; doi:10.1093/jxb/eru219)
Supplement: Supplementary Data [file supp_eru219_jexbot123513_file001.pdf]

Tomato PYR/PYL/RCAR ABA receptors show high expression in root, differential sensitivity to the ABA-agonist quinabactin and capability to enhance plant drought resistance

Miguel Gonzalez-Guzman[1*], Lesia Rodriguez[1*], Laura Lorenzo-Orts[1], Clara Pons[1], Alejandro Sarrion-Perdigones[1], Maria A. Fernandez[1], Marta Peirats-Llobet[1], Javier Forment[1], Maria Moreno-Alvero[2], Sean R. Cutler[3], Armando Albert[2], Antonio Granell[1] and Pedro L. Rodriguez[1†]

Table S1. List of oligonucleotides used in this work.

**Sl12g055990**	
ACCATGGTGAACAATATGGAAGATGAG	FNco12g055990
CTAGATTCTATCAATAGGGTCGGT	R12g055990
**Sl09g015380**	
ACCATGGCTCCAAAATCATCACTTTTAC	FNco09g015380
GGATCCTCATTTCAAATTTGTAGTATTTGAATTC	RBH09g015380
**Sl06g050500**	
ACCATGGCTTCTTCCCTTCAACTGCATC	FNco06g050500
TTAATCATGATCCATAGATAATTTATTAC	R06g050500
**Sl03g007310**	
ACCATGGACGCTAATGGATTCTGCGGTG	FNco03g007310
TTAGACCTGATCAATGGGTTCTG	R03g007310
**Sl08g76960**	
ACCATGGATAATAAACCGGAAACGTC	FNco08g76960
AAGCTTTCACCTGTGACTCGCATCACGAC	RHindIII08g076960
**Sl06g061180**	
ATGGAGCAATCCGATAACTCAAC	F06g061180
TTATGAGATCTCACCGTTACCACC	R06g061180
**Sl03g095780**	
ACCATGGCTTGTTCAGTTCAGCTGCAG	FNco03g095780
TCAGCGATTTGCAGTTGCGATTTG	R03g095780
**Sl12g096020**	
CGCAGTGTTTTTGAAGTGGACTAC	FΔN12g096020
TTATGAGGCCAATTGTGTTGAAG	R12g096020

<u>Solyc03g096670</u>

ACCATGGCTTTTGGCGAGACTAGAACT	FNco03g096670
CTATAGACCTCTTTTCAAATCAAC	R03g096670

<u>Solyc05g052980</u>

ATGGCTGGAATGTGTTGTGGAGT	F05g052980
TTATAGATTTTTTCTCAAATCCACC	R05g052980

<u>Solyc07g040990</u>

ACCATGGTTGTGGGTGCTGAAGATT	FNcoΔN7g040990
GTCGACTTATGTTTTCTTCTTGAATTTCCT	RSalI7g040990

<u>Solyc06g067980 (*SlLEA*)</u>

AGCAGATGTTGGAAAAGGAGC	FqPCR06g067980
ATGCCTATGGTGGGGTATGTG	RqPCRF06g067980

<u>Solyc02g084850 (*SlRAB18*)</u>

CCTGGGATGCATTGAACACC	FqPCR02g08450
CACGGGACACCATAACACAC	RqPCR02g08450

<u>Solyc06g019170 (*SlP5CS1*)</u>

ACCTTAATCTGGAGGCTTGAGAG	FqPCR06g019170
AATTATTTCACCCACCTGCCC	RqPCR06g019170

<u>Solyc06g009970 (*SlEF1a*)</u>

GACAGGCGTTCAGGTAAGGA	FqPCR06g009970
GGGTATTCAGCAAAGGTCTC	FqPCR06g009970

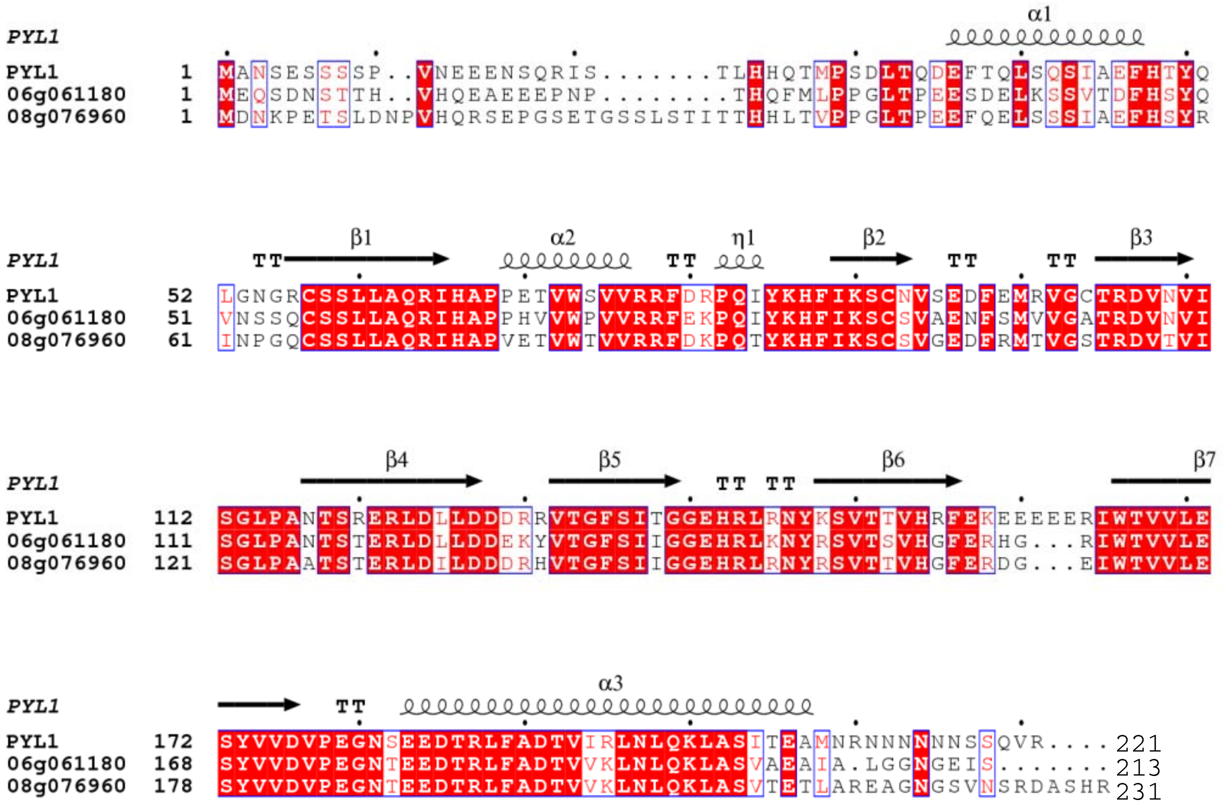

Fig. S1. Amino acid sequence alignment of AtPYL1 and putative tomato orthologous ABA receptors. The predicted secondary structure of the tomato proteins was depicted taking as model the crystallographic structure of AtPYL1 (Protein DataBank Code 3KAY) and using Espript interface (http://espript.ibcp.fr/).

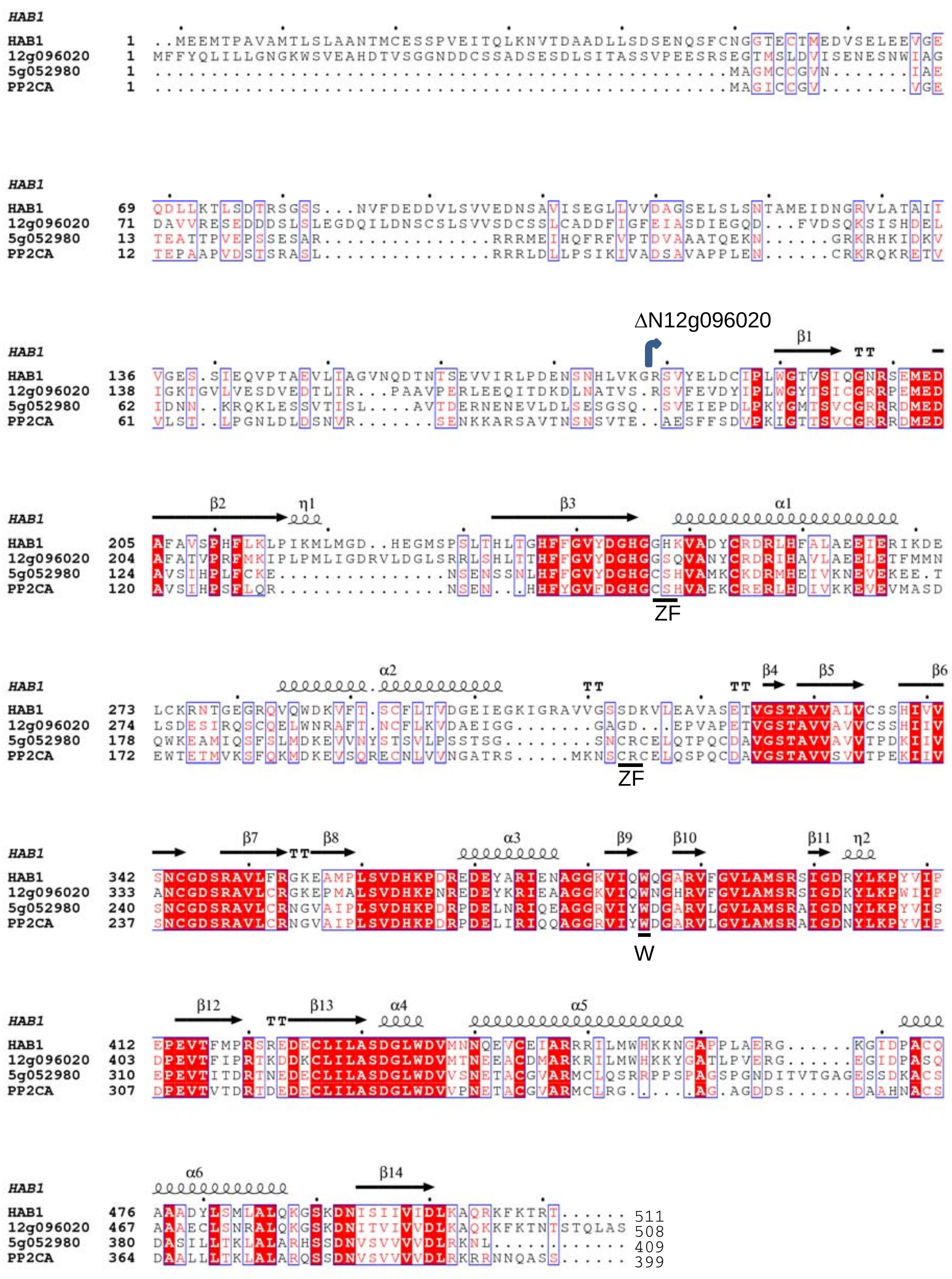

Fig. S2. Amino acid sequence alignment of AtHAB1, AtPP2CA and putative tomato orthologous PP2Cs. The predicted secondary structure of the tomato proteins was depicted taking as model the crystallographic structure of AtHAB1 (Protein DataBank Code 3QN1) and using Espript interface (http://espript.ibcp.fr/). The CCCH zing-finger (ZF) motif of AtPP2CA and 5g052980 and the tryptophan (W) conserved residue of clade A PP2Cs are underlined. The start of the ΔN 12g096020 PP2C is indicated.

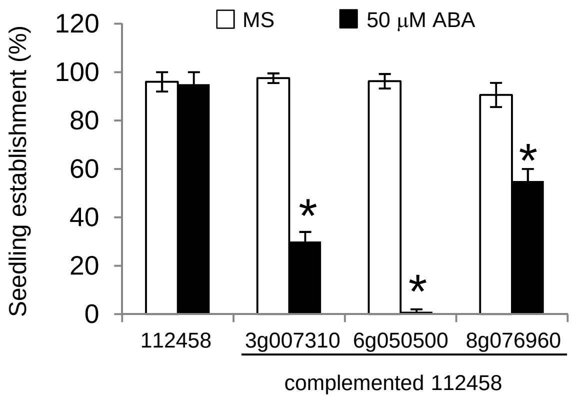

Fig. S3. Complementation of the 112458 *pyr/pyl* mutant by tomato PYR/PYL ABA receptors. Expression of HA-tagged 3g007310, 6g050500 or 8g076960 complements (partially in the case of 8g076960) the ABA-insensitive phenotype of 112458 *pyr/pyl* mutant, which lacks 6 major *Arabidopsis* ABA receptors, i.e. PYR1, PYL1, PYL2, PYL4, PYL5 and PYL8 (Gonzalez-Guzman et al., 2012). Approximately 100 seeds of each genotype (two independent experiments) were sown on MS plates lacking or supplemented with 50 µM ABA. Seedlings were scored for the presence of both green cotyledons and the first pair of true leaves after 8 d. Values are averages ± SE. * indicates p<0.05 (Student´s t test) when comparing data of 112458 mutant transformed with the indicated tomato receptor to 112458 in the same assay conditions.
